# Genome-wide identification and function analysis of *HMAD* gene family in cotton (*Gossypium* spp*.*)

**DOI:** 10.1186/s12870-021-03170-8

**Published:** 2021-08-20

**Authors:** Qinqin Wang, Xuke Lu, Xiugui Chen, Lanjie Zhao, Mingge Han, Shuai Wang, Yuexin Zhang, Yapeng Fan, Wuwei Ye

**Affiliations:** Institute of Cotton Research of Chinese Academy of Agricultural Sciences / Research Base, Zhengzhou University, State Key Laboratory of Cotton Biology / Key Laboratory for Cotton Genetic Improvement, MOA, Anyang, Henan 455000 China

**Keywords:** *HMAD* (heavy-metal-associated domain), Salt-stress, Cotton (*G. hirsutum*), Transcription factor

## Abstract

**Background:**

The abiotic stress such as soil salinization and heavy metal toxicity has posed a major threat to sustainable crop production worldwide. Previous studies revealed that halophytes were supposed to tolerate other stress including heavy metal toxicity. Though *HMAD* (heavy-metal-associated domain) was reported to play various important functions in *Arabidopsis*, little is known in *Gossypium*.

**Results:**

A total of 169 *G. hirsutum* genes were identified belonging to the *HMAD* gene family with the number of amino acids ranged from 56 to 1011. Additionally, 84, 76 and 159 *HMAD* genes were identified in each *G. arboreum, G. raimondii* and *G. barbadense*, respectively. The phylogenetic tree analysis showed that the *HMAD* gene family were divided into five classes, and 87 orthologs of *HMAD* genes were identified in four *Gossypium* species, such as genes *Gh_D08G1950* and *Gh_A08G2387* of *G. hirsutum* are orthologs of the *Gorai.004G210800.1* and *Cotton_A_25987* gene in *G. raimondii* and *G. arboreum*, respectively. In addition, 15 genes were lost during evolution. Furthermore, conserved sequence analysis found the conserved catalytic center containing an anion binding (CXXC) box. The *HMAD* gene family showed a differential expression levels among different tissues and developmental stages in *G. hirsutum* with the different cis-elements for abiotic stress.

**Conclusions:**

Current study provided important information about *HMAD* family genes under salt-stress in *Gossypium* genome, which would be useful to understand its putative functions in different species of cotton.

**Supplementary Information:**

The online version contains supplementary material available at 10.1186/s12870-021-03170-8.

## Background

Halophytes are ideal candidate crop for soil reclamation of heavy metal polluted soils [1]. Heavy metals (HMs), on the one hand, as micronutrient elements level (such as Fe, Cu, Zn, Co, Mn, Mo, Ni) is essential for the plant growth while become toxic in excess; on the other hand, other heavy metals (Ag^+^, Cd^2+^, Pb^2+^, Hg^2+^) even at low doses, are highly toxic because of no need for life and biological roles [2]. HMs contamination significantly affects not only the plant itself, but also the soil microbial community structure and function [3–5]. Heavy metal stress mainly concentrated in the signaling networks of calcium signaling, hormone signaling and MAPK (mitogen activated protein kinase) signaling and peroxide, which focused on ion detoxification and transport [6, 7]. Metal chelators is majorly Phytochelatins (PCs) and Metallothioneins (MTs), although MTs protects the plant from heavy metals by scavenging of the ROS and sequestration, even which is multi-resistant under abiotic stress such as cold, heat, salt, drought and so on [8, 9]. Compared to metal chelators, prominent groups of heavy metal ion transport families are P-type ATPases and the cation antiporters, such as *HMA* (Heavy metal ATPase), *ABC* (the ATP-binding cassette), *NRAMP* (Natural resistance and macrophage protein), *CDF* (Cation Diffusion Facilitator), yellow-stripe-like (YSL) transporter, *ZIP* (the Zrt, Irt-like proteins), *CAX* (the cation exchanger), *CTR* (the copper transporters), pleiotropic drug resistance (PDR) transporters, and metal responsive transcription factor 1 (MTF-1), distributing at plasma membrane or on tonoplast membrane of cell [10–14]. For *HMA* hyperaccumulators, vacuolar compartmentalization and HMs ion long-distance translocation that depends on P-type ATPases and a set of tonoplast transporters play important role in heavy metals homeostasis [15–17].

P-type ATPases have been subsided into 5 subfamilies, P1B ATPases (heavy metal pumps), P2A and P2B ATPases (Ca^2+^ pumps), P3A ATPases (plasma membrane H^+^ pumps), P4 ATPases (phospholipid-transporting ATPase) and P5 (no assigned specificity) subfamilies [18–20]. At least four P1B-ATPase subgroups with distinct metal selectivity: P1B-1 (include AtHMA5–8, OsHMA4–9), Cu^2+^, P1B-2 (include AtHMA2–4), Zn^2+^, P1B-3, Cu^2+^, P1B-4 (include AtHMA1), Co^2+^, which share a common catalytic mechanism with four important domains which are enzyme phosphorylation (P-domain), nucleotide binding (N-domain) and energy transduction (A-domain) and a transmembrane (TM) domain, respectively [21–23]. PIB-type ATPase lpg1024 (LpCopA) from *L. pneumophila* demonstrated that Cu^2+^ ion-entry path involves two ion-binding sites: one transient Met148-Cys382 site and one intramembranous site formed by trigonal coordination to Cys384, Asn689, and Met717 [24]. One nanobodies (Nbs) selected against the zinc-transporting PIB-2-ATPases ZntA from *Shigella sonnei* (SsZntA), significantly reduces the ATPase activity [25]. The multifunctional P1B-4-ATPase CzcP is part of the cobalt, zinc, and cadmium resistance system from the metal-tolerant, model organism *Cupriavidus metallidurans,* because of an evolutionarily adapted flexibility in the TM region likely afforded CzcP the ability to transport Cd^2+^ and Zn^2+^ in addition to Co^2+^ [26]. In *Mycobacterium tuberculosis*, replacement of the conserved Cys of P1B-4-ATPases at the metal binding pocket leads to a large reduction in Fe^2+^ but not Co^2+^ binding affinity [27]. In *Sphaerobacter thermophilus*, the P1B-1 and P1B-3-ATPase subfamilies both comprise Cu^2+^ transporters [28].

*HMA* (Heavy Metal ATPase) belonging to P1B-type ATPases (also called CPx-ATPases), is responsible for ion detoxification/transport [29–31] and vacuolar compartmentalization [32]. It is interesting in double mutant that *HMA* not only affects the transport of heavy metals [33], but also affect the plant growth and development [33]. And in rice, the DNA methylation state was altered in response to the heavy metal stress and showed transgenerational inheritance [34]. In *Sorghum bicolor*, arsenic stimulates expression of the P1B-ATPase transporter through the abscisic acid signaling pathway. In addition, Antioxidant Protein1 (*OsATX1*), as a Cu chaperone in rice, interacts with the P1B-ATPases *HMA4*, *HMA5*, *HMA6*, and *HMA9*, resulting in Cu trafficking and distribution in order to maintain Cu homeostasis in different rice tissues [35]. In a model of semi-halophyte *M. crystallinum*, *HMA4* (heavy metal ATPase 4) and *IRT2* (iron-regulated protein 2) had a significantly higher expression level compared to the control between Cd-untreated and NaCl-untreated, and effects on *IRT2* expression were cumulative [36]. Moreover, salinity stress overlaps with HMs toxicity to some extent, as several integrated mechanical and chemical signals are responsible for stress-related responses [37]. For example, chloroplast and chlorophyll content can measure salt stress [38], also affect the transport of heavy metals [39, 40]. Even flavonols have shown the ability in alleviating toxic effect of Pb and improving the resistance of plants, because it activated anti-oxidative process [41].

There are many similarities used as indicators for plant between heavy metal stress and salt stress, such as photosynthetic performance and stomatal behavior [42], photosynthetic pigment [43], proline [44, 45] and peroxidase [43]. ROS (reactive oxygen species) signal and the antioxidant system is a crosstalk among abiotic stresses, and the same for salt stress and heavy metal stress [46–50], which genes about peroxidase and GSH-AsA sysytem can not only improve salt tolerance, but also heavy metal tolerance [51–54]. Even genes associated with the GSH (glutathione) in sulfur metabolism enhance salt tolerance and heavy metals tolerance as well [55–58]. Furthermore, hormones alleviate salt stress and heavy metals stress, such as IAA (indole-3-acetic acid) [59, 60], Epibrassinolide [61, 62], Melatonin [63, 64], Ethylene-related gene [65, 66]. And the salicylic acid [67–69], NO [70–72], Silicon [73, 74] and biochar [75, 76] also can increase resistance to salt and heavy metal stresses.

Except the genes related with the antioxidant system, some genes responding to salt tolerance improve resistance to heavy metal stress. For example, a novel salt overly-sensitive 2 (SOS2) interaction protein SIP1 (SOS2 interaction protein 1) [77], the ubiquitin-specific protease (ZmUBP15, ZmUBP16 and ZmUBP19) [78], an ABA biogenesis inhibitor fluridone (FLUN) [79, 80], late embryogenesis abundant (LEA) or -related proteins [81, 82], Aquaporin (AQP) proteins [83, 84], plasma membrane H^+^-ATPase [85, 86], heat shock proteins [87, 88], a ramie bZIP transcription factor BnbZIP2 [89]. Some genes responding to heavy metal tolerance also enhance resistance to salt stress, such as phytochelatin synthase AtPCS2 [90], OsMT-3a (metallothionein-like type 3) [91]. Otherwise, some genes not reported to salt and heavy metal stresses can also improves salinity and heavy metal tolerance, for example, the pathogenesis-related protein [92–94], an ATP-binding cassette (ABC) transporter AtABCG36/AtPDR8 [95–97], CBS Domain Containing Protein OsCBSX4 [98], OsMIZ1 (MIZU-KUSSEI1) [99], OsSMP1 (stress membrane protein) [100].

The relationship between salt and heavy metals needs more research to show that the combined application of NaCl and CuSO_4_ has a significant adverse effect on wheat varieties [101]., while in cucumber (*Cucumis sativus* L.), salinity decreases the content of Zn uptake and increased other heavy metals (Cd, Cu, Ni, Pb) uptake [102]. What is more, there is an antagonistic effect of sodium chloride to differential heavy metal toxicity, especially to Cd^2+^ [103]. In *Spirodela polyrrhiza* (Lemnaceae), a high level of salinity inhibits the accumulation of the cadmium (Cd) and nickel (Ni) [104]. Ni at 20 mg kg-1 will increase the growth of wheat by alleviating salinity stress [105]. Additionally, Cd inhibited the Cu absorption of the root system [106], and cadmium was more toxic than copper on plants [107]. So far, the most researches about microorganisms have been reported both salt-tolerant and heavy-metal resistant, some of which can alleviate the heavy metal and salt stress in plants [108–111]. In addition, halophytes [112] and semi-halophyte [36] is known to be related to both salt and heavy metals. Besides, the eggplant breeding lines resistant against salt and drought stresses had higher Pb tolerance [113]. In willow species, *S. linearistipularis* had higher salt tolerance than *S. matsudana*, which plays important roles in heavy metal phytoextraction [106, 114].

Cotton (*Gossypium* L*.*), as a moderately salt-tolerant cash crop, is a pioneer crop for soil reclamation of saline-alkaline land [115, 116]. And cotton is an important fiber crop which provides the natural fiber for the textile industry [117]. Previously, much progress has been made in the identification of *HMAD* (heavy-metal-associated domain) genes in different plants [118–120]. However, there are no detail study has been reported in the identification, functional characterization, conserved domain analysis and expression profiles of the *HMAD* genes under salt-stress condition in cotton. The released genome sequence data of cotton and a publicly available database on CottonGen (https://www.cottongen.org/) allow us to comprehensively identify and analyze the *HMAD* gene family in cotton [117]. In this study, we conducted a comprehensive identification of *HMAD* genes in *G. hirsutum*, *G. barbadense*, *G. raimondii* and *G. arboreum*, with their chromosomal distribution, syntenic analysis, gene structure and conserved motifs analysis, as well as Ka/Ks values and expression pattern. In addition, predicted regulatory mechanism showed 111 *HMAD* genes were possibly regulated by salt-stress. This study will provide the basic information to explore the specific functions of *HMAD* gene family in cotton under salt-stress.

## Results

### Genome-wide identification and phylogenetic analysis

We used the Hidden Markov Model (HMM) profile of *HMAD* domain (PF00403) from Pfam (http://www.pfam.sanger.ac.uk/) database as queries to search the *HMAD* members in *G. hirsutum*, *G. arboreum*, *G. raimondii* and *G. barbadense* by Hmmer software with default parameters. A total of 169 proteins were identified belonging to the *HMAD* gene family in *G. hirsutum* with the number of amino acids ranged from 56 to 1011 (Table 1). Furthermore, we identified 84, 76 and 159 *HMAD* proteins in each *G. raimondii*, *G. arboreum* and *G. barbadense*, respectively (Table S1).

**Table 1:** *HMAD* genes in *Gossypium hirsutum*

GeneID	length (aa)	*pI*	*MV* (kDa)	*SL*	HMA Domain			TMHs	Signal peptide
					from	to	E-value		
Gh_A01G1069	69	8.2	7.5	chlo	2	61	1.20E-18	–	–
Gh_A01G1399	153	9.5	17	cyto	3	61	2.50E-12	–	–
Gh_A01G1576	150	10.2	16.5	chlo	4	61	1.10E-14	–	–
Gh_A01G1872	207	10.6	22.2	chlo	2	58	1.90E-12	–	1–25
Gh_A02G0496	302	5.8	33.3	nucl	2	59	3.70E-13	–	–
Gh_A02G1273	153	10	17	chlo	3	62	1.00E-14	–	–
Gh_A02G1652	155	9.3	17.5	cyto	3	61	1.10E-12	–	–
Gh_A03G0168	128	9.5	14.5	cyto	6	54	1.10E-09	–	–
Gh_A03G0250	307	9.7	33.3	nucl	9	57	5.10E-06	–	–
Gh_A03G0318	217	10.7	23.6	chlo	2	57	1.30E-13	–	–
Gh_A03G1525	956	8	101.1	plas	2	61	4.10E-10	7	–
Gh_A03G2159	166	10.7	18.6	chlo	2	58	2.00E-10	–	–
Gh_A04G0031	89	8.8	10.2	cyto	2	58	0.00036	–	–
Gh_A04G0056	192	6.3	22.2	chlo	2	62	6.70E-12	1	–
Gh_A04G0606	361	9	39.3	nucl	4	56	2.50E-11	–	–
Gh_A04G0674	290	10.5	31.1	chlo	2	48	6.30E-12	–	–
Gh_A04G0969	203	5.3	20.5	chlo	2	60	8.50E-10	–	–
Gh_A05G0151	133	8.1	14.9	chlo	1	58	4.90E-16	–	–
Gh_A05G0564	1000	6.3	108.5	plas	2	61	7.00E-10	8	–
Gh_A05G0838	223	10.3	24.2	chlo	1	57	2.00E-12	–	–
Gh_A05G0923	338	9.5	36.2	chlo	2	61	5.10E-08	–	–
Gh_A05G1306	143	8.4	16.6	nucl	3	61	7.30E-14	–	–
Gh_A05G1510	181	8.6	20.5	chlo	2	46	0.0011	–	–
Gh_A05G1511	161	9.8	18.1	chlo	2	46	3.10E-05	–	–
Gh_A05G1514	247	4.8	28.6	cyto	2	49	1.30E-09	–	–
Gh_A05G1975	150	10.1	16.7	chlo	4	56	7.00E-15	–	–
Gh_A05G2686	188	9.3	21.2	cyto	6	55	1.30E-10	–	–
Gh_A05G3385	110	5.5	13	cyto	25	61	5.40E-05	–	–
Gh_A05G3446	107	4.6	11.3	chlo	2	62	1.30E-17	–	–
Gh_A05G3792	475	6.9	53.9	nucl	4	59	1.30E-11	–	–
Gh_A06G0745	898	6.3	95.6	plas	2	61	9.70E-10	5	–
Gh_A06G1378	266	8.7	29.8	nucl	2	46	0.0026	–	–
Gh_A06G1738	254	5.4	28.9	chlo	3	54	3.80E-12	–	–
Gh_A07G0438	331	6.7	36.8	nucl	2	61	5.00E-13	–	–
Gh_A07G0686	149	10.1	16.7	cyto	3	61	1.30E-12	–	–
Gh_A07G0687	148	10.3	16.7	cyto	3	61	1.80E-14	–	–
Gh_A07G0866	146	10.6	15.9	nucl	2	58	1.20E-12	–	–
Gh_A07G0944	151	7.9	17.3	nucl	3	61	6.20E-17	–	–
Gh_A07G1285	197	8.6	22.5	cyto	6	58	2.50E-06	–	–
Gh_A07G1489	137	5	15.2	chlo	1	56	0.0013	–	–
Gh_A07G1505	237	7.3	26.6	cysk	2	62	5.10E-11	–	–
Gh_A07G2000	183	4.5	20	chlo	4	62	0.0033	–	–
Gh_A08G0091	74	10.1	8	chlo	1	57	5.20E-08	–	–
Gh_A08G0350	245	9.8	27.7	cyto	2	59	3.60E-08	–	–
Gh_A08G0681	295	5.7	31.6	nucl	2	61	2.20E-13	–	–
Gh_A08G0952	183	9.4	21.1	chlo	3	61	2.00E-11	1	1–22
Gh_A08G0990	112	9	12.4	cyto	17	61	4.50E-06	–	–
Gh_A08G1189	181	6.6	20.8	cyto	6	53	2.30E-06	–	–
Gh_A08G1589	336	7	35.5	chlo	2	62	2.60E-11	–	1–73
Gh_A08G1780	305	9.1	33.5	nucl	9	59	7.30E-07	–	–
Gh_A08G1851	348	6.9	38.2	nucl	3	62	5.10E-05	–	–
Gh_A08G1875	553	9.7	58.3	nucl	8	59	4.40E-12	–	–
Gh_A08G2388	987	6.4	107	plas	2	61	4.80E-13	8	–
Gh_A09G0406	104	8.9	11.6	cyto	2	61	4.10E-05	–	–
Gh_A09G0465	113	8.3	13.2	cyto	2	58	0.00021	–	–
Gh_A09G0709	139	7	16.1	nucl	3	61	7.10E-15	–	–
Gh_A09G1374	355	9.2	38.9	nucl	4	55	1.20E-09	–	–
Gh_A09G1682	182	10	20.9	cyto	4	61	5.00E-11	–	–
Gh_A09G1713	127	8.4	13.3	cyto	17	61	8.60E-07	–	–
Gh_A09G2192	329	5.2	37.2	nucl	3	54	3.00E-12	–	–
Gh_A10G1490	236	6.3	26.3	extr	7	59	5.20E-10	–	–
Gh_A10G1773	169	8.3	19	plas	2	62	5.30E-17	2	–
Gh_A10G2083	133	8.8	14.8	chlo	1	58	7.70E-16	–	–
Gh_A10G2291	100	8.5	11.3	cyto	7	61	4.30E-12	–	–
Gh_A11G1104	239	8.5	26.8	chlo	6	61	2.90E-08	–	–
Gh_A11G2390	522	8.9	54.2	nucl	8	62	3.80E-12	–	–
Gh_A11G2427	391	9.2	42	cyto	3	62	6.00E-12	–	–
Gh_A12G0038	185	9.9	20.6	nucl	9	59	2.80E-08	–	–
Gh_A12G0079	298	8.7	31.9	nucl	7	61	5.10E-13	–	–
Gh_A12G0443	1011	4.9	108.2	plas	2	62	2.20E-12	8	–
Gh_A12G0582	123	9.9	13.6	cyto	6	50	1.50E-06	–	–
Gh_A12G0960	182	6.8	20.7	chlo	6	59	1.60E-07	–	–
Gh_A12G1384	150	9.9	16.8	nucl	3	61	1.00E-13	–	–
Gh_A12G1537	121	10.5	13.6	nucl	4	42	0.00083	–	–
Gh_A12G1728	339	5.2	38.1	nucl	6	54	1.50E-10	–	–
Gh_A12G2078	177	10.5	20	cyto	2	61	3.30E-05	–	–
Gh_A12G2194	142	9.1	16.5	cyto	3	61	1.80E-11	–	–
Gh_A12G2219	340	4.8	38.2	cyto	2	58	3.00E-08	–	–
Gh_A12G2220	133	9.8	15.5	chlo	6	39	3.40E-07	–	–
Gh_A12G2296	144	8.4	15.9	chlo	11	54	0.00078	–	–
Gh_A12G2297	125	8.7	13.9	cyto	7	56	5.00E-07	–	–
Gh_A12G2326	151	6.5	17.1	cyto	2	61	3.20E-13	–	–
Gh_A12G2525	150	10.9	16.8	chlo	2	57	1.30E-12	–	–
Gh_A13G0122	70	7.3	7.9	chlo	3	61	4.60E-12	–	–
Gh_A13G2272	243	10.5	26.6	chlo	2	48	2.90E-13	–	–
Gh_D01G0091	244	8.5	27.9	nucl	9	61	7.80E-05	–	–
Gh_D01G1151	75	9.3	8.1	chlo	2	62	1.70E-12	–	–
Gh_D01G1640	127	9.4	14	chlo	3	61	1.70E-12	–	–
Gh_D01G1883	150	10.2	16.5	chlo	4	61	1.10E-14	–	–
Gh_D01G2129	197	10.4	21.1	chlo	2	58	1.10E-12	–	1–25
Gh_D02G0556	300	5.6	33.1	nucl	2	59	3.60E-13	–	–
Gh_D02G1991	211	10.9	24.2	chlo	2	58	2.80E-13	–	–
Gh_D03G0070	155	9.1	17.6	cyto	3	61	3.80E-14	–	–
Gh_D03G0414	157	10	17.7	chlo	3	62	1.10E-14	–	–
Gh_D03G1260	217	10.7	23.6	chlo	2	57	1.30E-13	–	–
Gh_D03G1316	333	10.1	36.6	cyto	9	57	1.70E-06	1	–
Gh_D03G1416	152	9.6	17.4	cyto	23	54	0.014	1	–
Gh_D04G0001	308	4.8	34.3	cyto	4	55	1.20E-08	–	–
Gh_D04G0145	121	4.5	12.8	chlo	2	62	1.80E-17	–	–
Gh_D04G0199	129	5.5	15.1	cyto	25	61	0.00046	–	–
Gh_D04G1066	321	9	34.7	nucl	11	58	7.00E-08	–	–
Gh_D04G1139	290	10.5	31.1	chlo	2	48	6.30E-12	–	–
Gh_D04G1512	199	5	20.3	chlo	2	42	3.90E-09	–	–
Gh_D05G0215	133	8.1	14.9	chlo	1	58	4.90E-16	–	–
Gh_D05G0693	991	6.3	107.4	plas	2	61	1.30E-10	8	–
Gh_D05G1007	338	9.5	36.1	cyto	2	61	5.10E-08	–	–
Gh_D05G1684	178	8.6	19.8	chlo	2	57	0.00026	–	–
Gh_D05G1685	258	6	29.4	nucl	3	49	3.10E-09	–	–
Gh_D05G2202	150	10.1	16.6	chlo	4	56	2.70E-15	–	–
Gh_D05G2984	224	9.2	25.2	cyto	6	58	1.70E-09	–	–
Gh_D05G3677	174	6.1	19.7	cyto	2	62	3.90E-12	–	–
Gh_D05G3899	223	10.6	24.4	chlo	1	57	1.50E-12	–	–
Gh_D06G0881	898	6.6	95.8	plas	2	61	1.10E-09	5	–
Gh_D06G2239	255	5.4	28.9	chlo	3	54	3.80E-12	–	–
Gh_D07G0041	807	9	89	plas	1	58	1.50E-14	10	–
Gh_D07G0501	320	7.4	35.4	nucl	3	55	8.20E-11	–	–
Gh_D07G0768	149	10.2	16.7	cyto	3	61	1.10E-12	–	–
Gh_D07G0769	143	10.3	16.2	cyto	4	61	1.30E-14	–	–
Gh_D07G0938	147	10.8	16.3	chlo	3	58	3.10E-10	–	–
Gh_D07G1023	145	8	16.6	nucl	3	61	8.70E-17	–	–
Gh_D07G1399	172	8.6	19.8	cyto	6	58	1.90E-06	–	–
Gh_D07G1640	136	5.3	15.2	chlo	1	56	0.0015	–	–
Gh_D07G1743	237	6.9	26.8	cysk	2	62	5.10E-11	–	–
Gh_D08G0132	65	8.8	7.1	chlo	7	55	0.0027	–	–
Gh_D08G0133	74	9.9	7.9	chlo	2	57	4.20E-07	–	–
Gh_D08G0448	234	9.9	26.4	cyto	2	59	1.00E-09	–	–
Gh_D08G0789	294	6.7	31.7	nucl	2	61	1.90E-14	–	–
Gh_D08G1161	183	9.4	21.2	chlo	3	61	2.00E-11	1	1–22
Gh_D08G1263	112	9	12.5	cyto	17	61	4.50E-06	–	–
Gh_D08G1473	181	5.5	20.8	chlo	6	53	6.20E-06	–	–
Gh_D08G1899	329	6.9	34.9	chlo	2	62	3.20E-12	–	1–65
Gh_D08G1950	817	7.2	89.1	plas	2	61	3.80E-13	6	–
Gh_D08G2126	305	9.4	33.4	nucl	8	60	9.70E-09	–	–
Gh_D08G2212	348	6.9	38	nucl	3	62	6.40E-05	–	–
Gh_D08G2237	544	9.6	57.2	nucl	8	59	2.40E-12	–	–
Gh_D09G0421	102	8.9	11.4	cyto	2	61	3.90E-05	–	–
Gh_D09G0474	113	8.3	13.2	cyto	2	58	0.00021	–	–
Gh_D09G0521	511	8.6	54.1	chlo	2	62	2.20E-11	–	–
Gh_D09G1375	351	8.8	38.7	nucl	4	55	1.10E-09	–	–
Gh_D09G1777	182	10	20.9	cyto	4	61	5.00E-11	–	–
Gh_D09G1816	125	8.4	13.2	cyto	17	54	1.40E-06	–	–
Gh_D09G2471	137	8	15.9	nucl	3	61	1.10E-15	–	–
Gh_D10G0078	250	9.3	27.5	nucl	2	43	0.00023	–	–
Gh_D10G1733	231	6.1	25.8	cyto	3	59	3.40E-11	–	–
Gh_D10G2047	82	8.2	8.7	chlo	2	62	8.40E-18	–	–
Gh_D11G2705	475	8	49.5	nucl	8	61	8.50E-12	–	–
Gh_D11G2744	391	9.1	42	nucl	3	62	6.00E-12	–	–
Gh_D12G0095	297	8.5	31.8	nucl	7	61	5.10E-13	–	–
Gh_D12G0134	164	11	18.5	chlo	2	58	5.20E-13	–	–
Gh_D12G0431	121	8.6	13.5	chlo	14	59	0.0024	–	–
Gh_D12G0446	1011	4.9	108.1	plas	2	62	1.10E-12	8	–
Gh_D12G0594	123	9.9	13.6	cyto	4	50	1.10E-06	–	–
Gh_D12G1072	183	6.8	20.8	cyto	6	59	1.60E-07	–	–
Gh_D12G1507	150	9.9	16.8	nucl	3	61	1.00E-13	–	–
Gh_D12G1886	336	5.2	37.9	cyto	6	54	6.90E-12	–	–
Gh_D12G2164	106	9.9	12.1	chlo	2	43	3.70E-07	1	–
Gh_D12G2254	98	10	10.9	cyto	2	61	5.80E-05	–	–
Gh_D12G2256	76	9.5	8.4	cyto	7	61	2.70E-05	–	–
Gh_D12G2374	180	9.6	20.9	cyto	3	61	2.60E-11	–	–
Gh_D12G2433	245	7.9	27	chlo	8	54	6.60E-06	–	–
Gh_D12G2434	129	9.1	14.6	cyto	6	56	0.00032	–	–
Gh_D12G2460	151	6.5	17.1	cyto	2	61	3.20E-13	–	–
Gh_D12G2725	340	4.9	38.2	cyto	2	58	3.00E-08	–	–
Gh_D12G2726	133	9.8	15.6	chlo	6	41	1.80E-08	–	–
Gh_D13G0138	70	7.3	7.9	chlo	3	61	4.60E-12	–	–
Gh_D13G1000	56	10.7	5.8	chlo	23	49	8.10E-05	–	–
Gh_Sca004952G01	329	4.9	37.4	cyto	3	54	1.10E-11	–	–
Gh_Sca011408G01	127	9.4	14	chlo	3	61	1.70E-12	–	–
Gh_Sca013298G01	308	4.8	34.3	cyto	4	55	1.20E-08	–	–

**Fig. 1:**
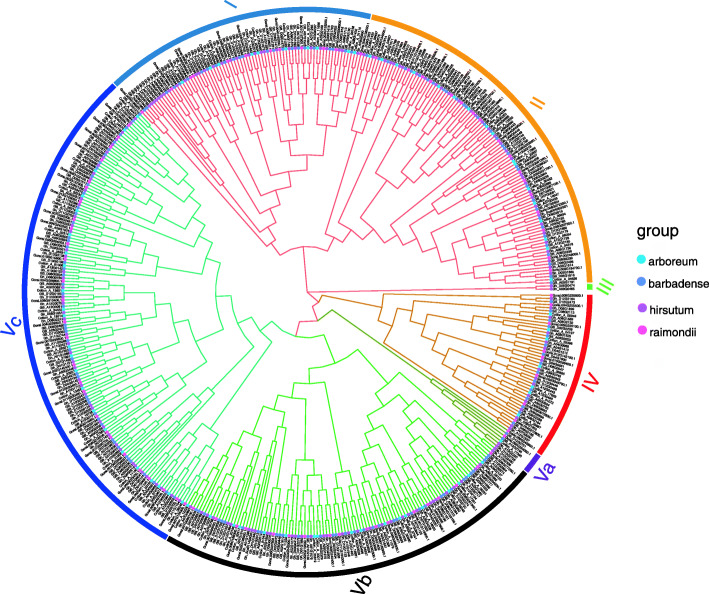
A phylogenetic tree of *HMAD* genes in four *Gossypium* species. Five clades (I, II, III, IV, Va, Vb, Vc) of *HMAD* family genes presented between *G. arboreum*, *G.barbadense*, *G.hirsutum* and *G.raimondii* were emphasized in different colors using ggtree (v2.2.4) packages of R (v4.0.3) software

In order to explore the evolutionary relationships of the *HMAD* gene family, an unrooted phylogenetic tree was constructed using the full length *HMAD* protein sequences from *G. arboreum*, *G. barbadense, G. raimondii*, *G. hirsutum* (Fig. 1). The *HMAD* proteins in the four *Gossypium* species were divided into five groups (I, II, III, IV, Va, Vb, Vc), which the P1B-ATPases *HMA5–8* belongs to IV group (Table S3). Additionally, 87 orthologs of *HMAD* genes (Table 2) were identified in four *Gossypium* species (I account for 18.39%, II account for 18.39%, III account for 1.15%, IV account for 10.34%, Va account for 1.15%, Vb account for 20.69%, Vc account for 29.89%) (Fig. 1), such as genes *Gh_D08G1950* and *Gh_A08G2387* of *G. hirsutum* are orthologs of the *Gorai.004G210800.1* and *Cotton_A_25987* gene in *G. raimondii* and *G. arboreum*, respectively.

**Table 2:** The Ka and Ks values of homologous pairs

GeneID	GeneID	Ka	Ks	ka/ks
Gh_D08G1950	Gh_A08G2387	2.89	0.51	5.63
Gh_D05G0215	Gh_D07G0041	0.50	0.10	4.86
Gh_A10G2083	Gh_D07G0041	0.23	0.05	4.78
Gh_D07G0041	Gh_A10G2084	0.05	0.04	1.45
Gh_A08G1875	Gh_D08G2237	0.01	0.01	1.20
Gh_A12G0079	Gh_D12G0095	0.02	0.02	1.19
Gh_A05G2686	Gh_D05G2984	0.05	0.05	1.18
Gh_A11G1104	Gh_D11G1254	0.14	0.12	1.17
Gh_A12G0038	Gh_D12G0053	0.02	0.02	1.16
Gh_A07G0866	Gh_D07G0938	0.13	0.11	1.14
Gh_A13G2272	Gh_D13G0999	0.04	0.03	1.13
Gh_A08G0091	Gh_D08G0133	0.02	0.02	1.10
Gh_A10G1490	Gh_D10G1733	0.02	0.02	1.08
Gh_A11G1104	Gh_A12G0038	0.33	0.35	0.95
Gh_A04G0969	Gh_D04G1512	0.03	0.03	0.91
Gh_A01G1872	Gh_D01G2129	0.01	0.01	0.90
Gh_A12G2078	Gh_D12G2254	0.05	0.06	0.90
Gh_D12G2434	Gh_A12G2298	0.19	0.22	0.86
Gh_A04G0056	Gh_D05G3677	0.06	0.07	0.85
Gh_A02G1273	Gh_D03G0414	0.14	0.18	0.79
Gh_A05G3792	Gh_D05G1830	0.01	0.02	0.76
Gh_A05G1510	Gh_A10G0074	0.48	0.65	0.74
Gh_A10G1773	Gh_D10G2047	0.04	0.06	0.73
Gh_A03G0250	Gh_D03G1316	0.11	0.15	0.71
Gh_A12G2296	Gh_D12G2433	0.05	0.07	0.70
Gh_A08G0350	Gh_D08G0448	0.04	0.06	0.70
Gh_A08G0681	Gh_D08G0789	0.03	0.05	0.67
Gh_A12G2297	Gh_D12G2434	0.11	0.19	0.61
Gh_A11G2390	Gh_D11G2705	0.01	0.02	0.59
Gh_A05G1306	Gh_D05G1476	0.02	0.04	0.57
Gh_A03G0168	Gh_D03G1416	0.01	0.02	0.55
Gh_A08G1851	Gh_D08G2212	0.01	0.02	0.55
Gh_D08G0789	Gh_D12G0095	0.24	0.45	0.53
Gh_A12G2525	Gh_D12G0134	0.01	0.03	0.53
Gh_A08G1189	Gh_D08G1473	0.01	0.02	0.53
Gh_A08G1780	Gh_D08G2126	0.01	0.02	0.52
Gh_A08G0681	Gh_A12G0079	0.24	0.47	0.51
Gh_A05G0838	Gh_D05G3899	0.02	0.03	0.49
Gh_A05G1514	Gh_D05G1685	0.04	0.09	0.46
Gh_A05G1510	Gh_D05G1684	0.14	0.31	0.44
Gh_A08G1589	Gh_D08G1899	0.02	0.04	0.44
Gh_A08G0990	Gh_D08G1263	0.01	0.03	0.42
Gh_D07G1640	Gh_D11G1512	0.23	0.56	0.41
Gh_A04G0606	Gh_D04G1066	0.03	0.07	0.40
Gh_A08G2388	Gh_D08G1950	0.02	0.04	0.40
Gh_A07G1489	Gh_D07G1640	0.02	0.06	0.40
Gh_A06G1378	Gh_A10G0074	0.22	0.57	0.39
Gh_D10G0078	Gh_A10G0074	0.06	0.15	0.39
Gh_D05G1684	Gh_D10G0078	0.26	0.69	0.38
Gh_A11G2427	Gh_D11G2744	0.02	0.06	0.36
Gh_A07G1505	Gh_D07G1743	0.01	0.04	0.35
Gh_A07G2000	Gh_D07G2221	0.02	0.04	0.35
Gh_A07G1489	Gh_A11G1367	0.22	0.64	0.35
Gh_A06G0745	Gh_D06G0881	0.01	0.03	0.34
Gh_A07G0944	Gh_D07G1023	0.02	0.06	0.34
Gh_A03G0318	Gh_A07G0866	0.19	0.57	0.34
Gh_A03G2159	Gh_D02G1991	0.01	0.02	0.34
Gh_D03G1260	Gh_D07G0938	0.19	0.57	0.33
Gh_A05G0564	Gh_D05G0693	0.01	0.02	0.33
Gh_A07G1285	Gh_D07G1399	0.02	0.05	0.33
Gh_D08G1161	Gh_D03G0822	0.09	0.29	0.32
Gh_A12G0582	Gh_D12G0594	0.01	0.02	0.30
Gh_D09G0521	Gh_A09G0524	0.02	0.06	0.30
Gh_A08G0952	Gh_D08G1161	0.00	0.01	0.30
Gh_A03G0318	Gh_A05G0838	0.16	0.53	0.29
Gh_A02G1652	Gh_D03G0070	0.02	0.07	0.29
Gh_A08G1875	Gh_A11G2390	0.13	0.44	0.28
Gh_A05G0923	Gh_A08G1780	0.14	0.49	0.28
Gh_D02G1991	Gh_D12G0134	0.14	0.49	0.28
Gh_D12G2254	Gh_A12G2080	0.03	0.12	0.28
Gh_A03G0250	Gh_A08G1780	0.15	0.55	0.27
Gh_A09G0709	Gh_D09G2471	0.01	0.03	0.27
Gh_A07G0438	Gh_A09G2192	0.17	0.64	0.27
Gh_A09G1374	Gh_D09G1375	0.02	0.07	0.27
Gh_D05G1007	Gh_D08G2126	0.14	0.51	0.27
Gh_A05G3446	Gh_D04G0145	0.02	0.06	0.27
Gh_A05G1510	Gh_A06G1378	0.15	0.59	0.26
Gh_D08G2237	Gh_D11G2705	0.12	0.44	0.26
Gh_A03G0250	Gh_A05G0923	0.14	0.54	0.26
Gh_A12G0960	Gh_D12G1072	0.00	0.01	0.26
Gh_A08G0952	Gh_A02G0943	0.08	0.31	0.25
Gh_A09G0465	Gh_D09G0474	0.00	0.02	0.23
Gh_A12G1728	Gh_D12G1886	0.01	0.07	0.23
Gh_D07G0501	Gh_D12G1886	0.19	0.86	0.22
Gh_A03G0318	Gh_D03G1260	0.00	0.02	0.21
Gh_A06G1378	Gh_D06G1721	0.02	0.10	0.21
Gh_A02G0496	Gh_D02G0556	0.01	0.05	0.20
Gh_A04G0674	Gh_D04G1139	0.01	0.03	0.20
Gh_D10G0078	Gh_D06G1721	0.21	1.03	0.20
Gh_A07G0438	Gh_A12G1728	0.18	0.91	0.20
Gh_A07G0438	Gh_D07G0501	0.01	0.04	0.18
Gh_A12G2194	Gh_D12G2374	0.00	0.02	0.18
Gh_A07G0686	Gh_D07G0768	0.01	0.05	0.17
Gh_A12G0443	Gh_D12G0446	0.01	0.04	0.17
Gh_A09G2192	Gh_A12G1728	0.12	0.75	0.16
Gh_A06G1738	Gh_D06G2239	0.01	0.06	0.16
Gh_A01G1069	Gh_D01G1151	0.01	0.08	0.15
Gh_A09G2192	Gh_D12G1886	0.12	0.80	0.15
Gh_A07G0687	Gh_D07G0769	0.01	0.04	0.15
Gh_A12G1537	Gh_D12G1670	0.00	0.02	0.15
Gh_A05G0923	Gh_D05G1007	0.01	0.05	0.14
Gh_A04G0606	Gh_A09G1374	0.07	0.50	0.14
Gh_A05G1975	Gh_D05G2202	0.01	0.06	0.13
Gh_A09G0406	Gh_D09G0421	0.00	0.03	0.13
Gh_D01G1883	Gh_D05G2202	0.04	0.45	0.09
Gh_A12G2219	Gh_D12G2725	0.00	0.04	0.09
Gh_A13G0122	Gh_D13G0138	0.01	0.07	0.09
Gh_A05G3385	Gh_D04G0199	0.00	0.05	0.08
Gh_A12G2326	Gh_D12G2460	0.00	0.05	0.06
Gh_A09G1682	Gh_D09G1777	0.00	0.05	0.05
Gh_A09G1713	Gh_D09G1816	0.00	0.10	0.03
Gh_A01G1399	Gh_D01G1640	0	0.02	0
Gh_A05G0151	Gh_D05G0215	0	0.05	0
Gh_A12G1384	Gh_D12G1507	0	0.02	0
Gh_A01G1576	Gh_D01G1883	0.01	0	
Gh_A12G2220	Gh_D12G2726	0.00	0	

### Chromosomal distribution and syntenic analysis

**Fig. 2:**
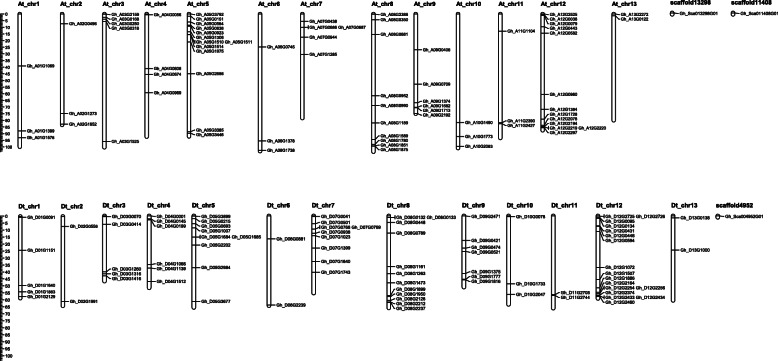
Mapping of the *HMAD* genes in the chromosomes. Partial *HMAD* genes localized in scaffolds. White color bar indicated the chromosomes from At and Dt sub genomes of *G. hirsutum*. At_chr1-At_chr13 represented the chromosomes from At sub genome while Dt_chr1-Dt_chr13 represented the chromosomes from Dt sub genome. Genes’ chromosomal locations calculated from published genome data were presented at the left side of each chromosome of At and Dt sub genome; and corresponding gene names were written at the right of each chromosome of At and Dt sub genome

**Fig. 3:**
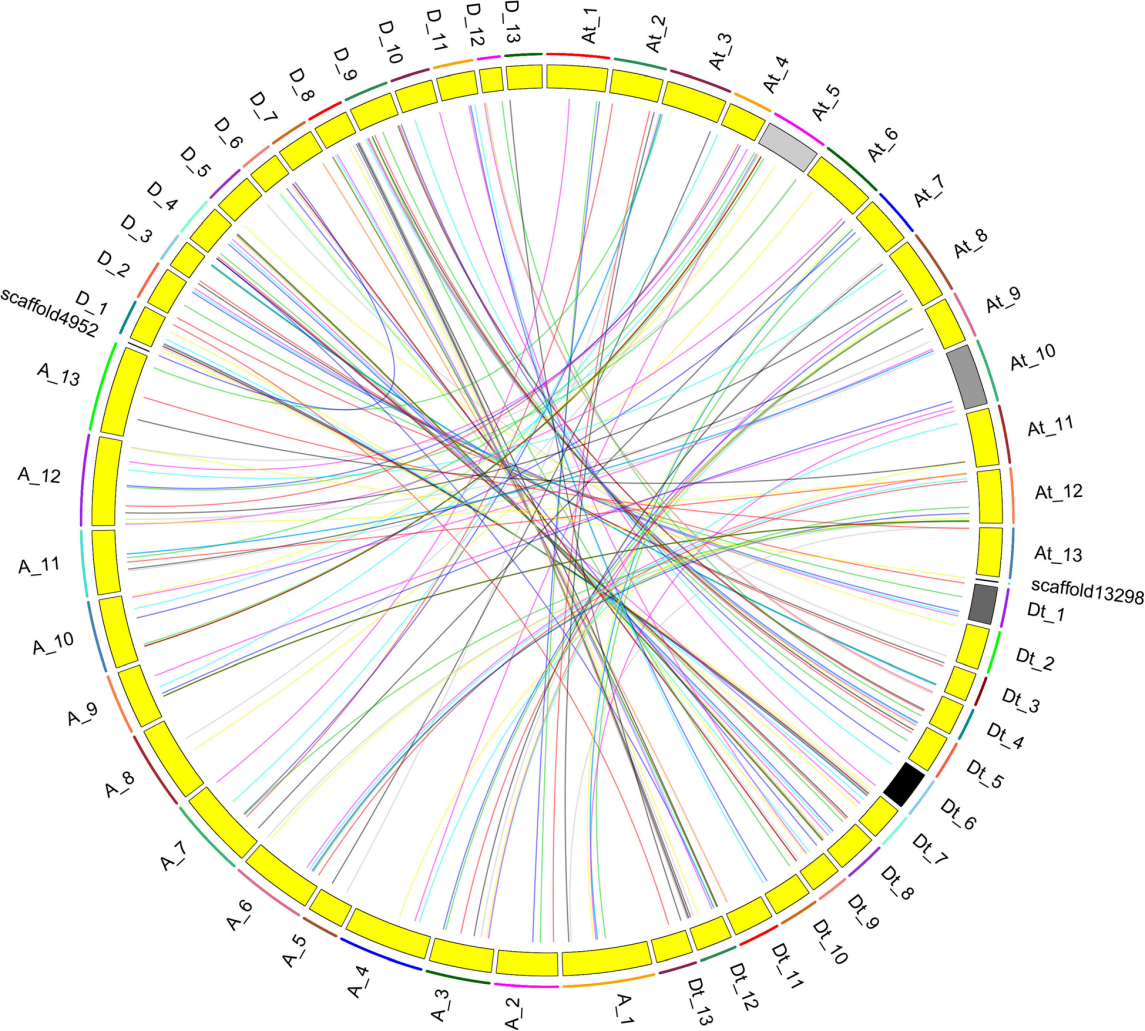
Genome wide synteny analysis of *HMAD* genes between *G. hirsutum* and two diploid cotton species. Collinearity analysis of *G. hirsutum* (At and Dt) in *G. arboreum* (A) and *G. raimondii* (D) genomes. The syntenics were connected by different colored lines. *G. hirsutum* chromosomes were separated into At and Dt chromosomes. Each genome’s chromosomes were displayed in different colors

Physical mapping of the 169 *G. hirsutum HMAD* genes showed that 79 and 77 *HMAD* genes were variably distributed on 26 chromosomes of the A and D sub-genomes, respectively (Fig. 2), among which 13 genes localized in scaffold. Additionally, a maximum of 17 and 16 genes were localized on the paralogous chromosome 12 of the A sub-genomes and D sub-genomes. Moreover, there were nine pairs and two gene clusters were marked as tandem duplication based on the criteria of less than five intervening genes. Among these tandem duplication genes, five pairs and two clusters belonged to group Vb except of *Gh_D05G1684* - *Gh_D05G1685* and *Gh_A05G1510* - *Gh_A05G1511*pairs, which belonged to group III. To study the locus relationship of orthologs between the A and D sub-genomes, we also performed synteny analysis. 72 and 73 *HMAD* genes were unevenly mapped onto 13 chromosomes of *G. arboreum* and *G. raimondii*, respectively. In *G. arboreum*, each chromosome contained 2–11 *HMAD* members. Chromosome 12 contained 11 *HMAD* genes, while chromosome 5 and chromosome 8 had two *HMAD* genes, respectively. And one gene of *G. arboreum* on chromosome 12 correspond to *Gh_Sca013298G01* in scaffold13298 (Fig. 3). In *G. raimondii*, the number of each chromosome genes ranged from 1 to 15 *HMAD* members. Chromosome 8 contained maximum 15 *HMAD* genes, while chromosome 13 had only one *HMAD* gene. Otherwise, one gene of *G. raimondii* on chromosome 6 correspond to *Gh_Sca004952G01* in scaffold4952 (Fig. 3). The result of synteny analysis indicated that most of the *HMAD* genes loci were highly conserved between the A and D sub-genomes respectively (Fig. 3), and 15 genes were lost during evolution, among which 4 in A genome (*Cotton_A_04626*, *Cotton_A_25931*, *Cotton_A_00150*, *Cotton_A_35231*), 11 in D genome (*Gorai.001G250300.1*, *Gorai.005G218500.1*, *Gorai.005G220100.1*, *Gorai.007G134300.1*, *Gorai.007G295300.1*, *Gorai.008G005700.1*, *Gorai.009G162900.1*, *Gorai.009G199900.1*, *Gorai.009G414800.1*, *Gorai.012G027800.1*, *Gorai.008G245900.1*). We surveyed the collinear relationship among the orthologous *HMAD* genes between *G. barbadense* and *G. hirsutum* (Fig. S2). There were 161 pair genes in *G. barbadense* and *G. hirsutum*. In *G. barbadense*, 154 genes (except *GB_A01G1916*, *GB_A03G2039*, *GB_A04G0061*, *GB_A12G2848*, *GB_D05G3226*, *GB_D07G1125*, *GB_D12G2855*) showed the correspondent relationship among *HMAD* gene family from A-subgenome, D-subgenome respectively between *G. barbadense* and *G hirsutum* (Fig. S2), and 5 genes (*GB_A09G0824*, *GB_D05G1602*, *GB_D05G1968*, *GB_D07G0037*, *GB_D12G0056*) were not found the correspondent relationship to *HMAD* gene family of *G. hirsutum*. To *G. hirsutum*, 153 genes (except *Gh_A03G1525*, *Gh_A03G2159*, *Gh_A05G1511*, *Gh_A10G2291*, *Gh_D08G2126*, *Gh_D12G2433*, *Gh_A08G0952* with three correspondent relationship) showed the correspondent relationship among *HMAD* gene family from A-subgenome, D-subgenome respectively between *G. hirsutum* and *G. barbadense* (Fig. S2), and 16 genes (*Gh_A04G0031*, *Gh_A05G1510*, *Gh_A06G1378*, *Gh_A07G1489*, *Gh_A07G2000*, *Gh_A09G0465*, *Gh_A11G1104*, *Gh_A12G1537*, *Gh_D05G1684*, *Gh_D07G0041*, *Gh_D07G1640*, *Gh_D08G0132*, *Gh_D09G0474*, *Gh_D10G0078*, *Gh_D12G0431*, *Gh_D12G2254*) were not found the correspondent relationship to *HMAD* gene family of *G. barbadense*. We also found that the *HMAD* genes located on A02 and A03 chromosomes while their corresponding orthologs were located on D03 and D02 (Table 2), respectively. These results are consistent with the previous research [121], which might be due to the chromosomal translocation between Chr02 and Chr03 before cotton polyploidization forming an allotetraploid [121].

### Analysis of gene structure and conserved motifs

**Fig. 4:**
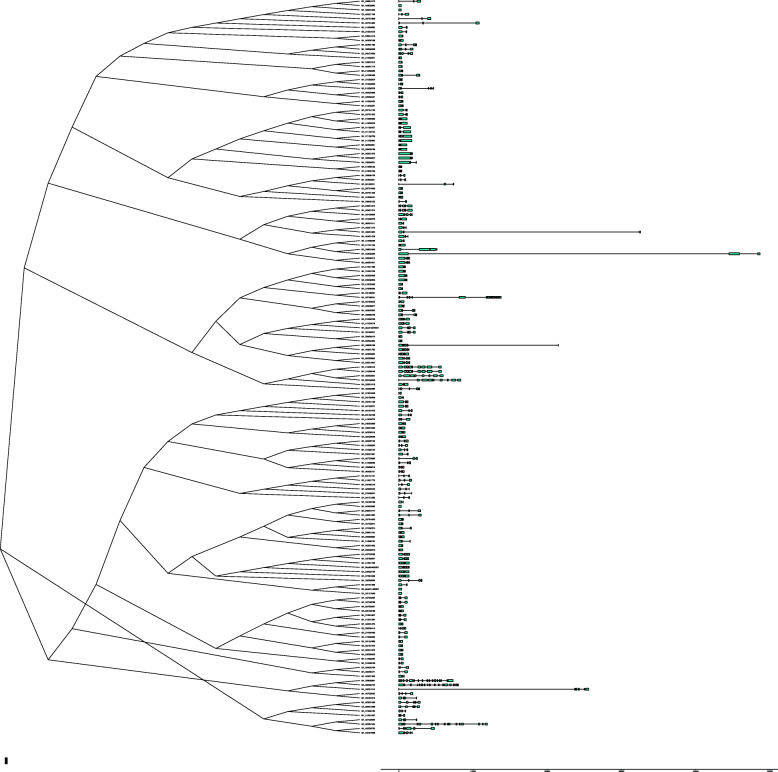
Gene structures of *HMAD* genes in *G. hirsutum*. A phylogenetic tree from HMAD protein sequences was constructed with MEGA X using neighbor-joining method. Green blocks and black lines represented exon and intron positions, respectively. And the scale bar is present at the bottom

Gene structure is important to determine its role in showing the phylogenetic relation between the *HMAD* genes. A NJ tree was generated with MEGA using all the *HMAD* protein sequences from *G. hirsutum* and gene structure was determined (Fig. 4). As shown in the Fig. 4, *HMAD* genes from *G. hirsutum* were divided into five subclades (group I, group II, group III, group IV, group Va and group Vb, among which, group I contained 13 genes while group II to group Va and group Vb contained 66, 29, 14, 22 and 25 genes, respectively. Furthermore, the analysis of gene structure showed that the introns in the gene structure are particularly variable among of *HMAD* gene family, which include 5 genes (*Gh_D01G1640*, *Gh_Sca011408G01*, *Gh_A05G3385* of group I, *Gh_D08G1263* and *Gh_A08G0990* of group Vb) without intron, 35 genes with 1 intron, 79 genes with 2 introns, 23 genes with 3 introns, 17 genes with 4 introns, one gene (*Gh_A05G0564* belonging to P1B-ATPases *HMA5*) with 7 introns, 3 genes (*Gh_D05G0693* belonging to P1B-ATPases *HMA5*, *Gh_A12G0443* belonging to P1B-ATPases *HMA7*, *Gh_D12G0446* belonging to P1B-ATPases *HMA7*) with 8 introns, one gene (*Gh_D07G0041*) with 15 introns, 3 genes (*Gh_D06G0881* belonging to P1B-ATPases *HMA8*, *Gh_A06G0745* belonging to P1B-ATPases *HMA8* and *Gh_A03G1525* belonging to P1B-ATPases *HMA6*) with 16 introns. *Gh_D06G0881* and *Gh_A06G0745* was divided into cluster I between the four *Gossypium* species (Fig. 1), and in *G. hirsutum* (Fig. 4). *Gh_A03G1525* was divided into cluster 1 between the four *Gossypium* species (Fig. 1), whereas it was grouped into cluster II in *G. hirsutum* (Fig. 4). *Gh_A12G0443*, *Gh_D12G0446*, *Gh_D05G0693*, *Gh_A05G0564* was divided into cluster I between the four *Gossypium* species (Fig. 1), whereas it was grouped into cluster III in *G. hirsutum* (Fig. 4). Though the number of genes used for generating this phylogenetic tree was different from the phylogenetic tree shown in Fig. 1, the gene members within the subclades were nearly same.

**Fig. 5:**
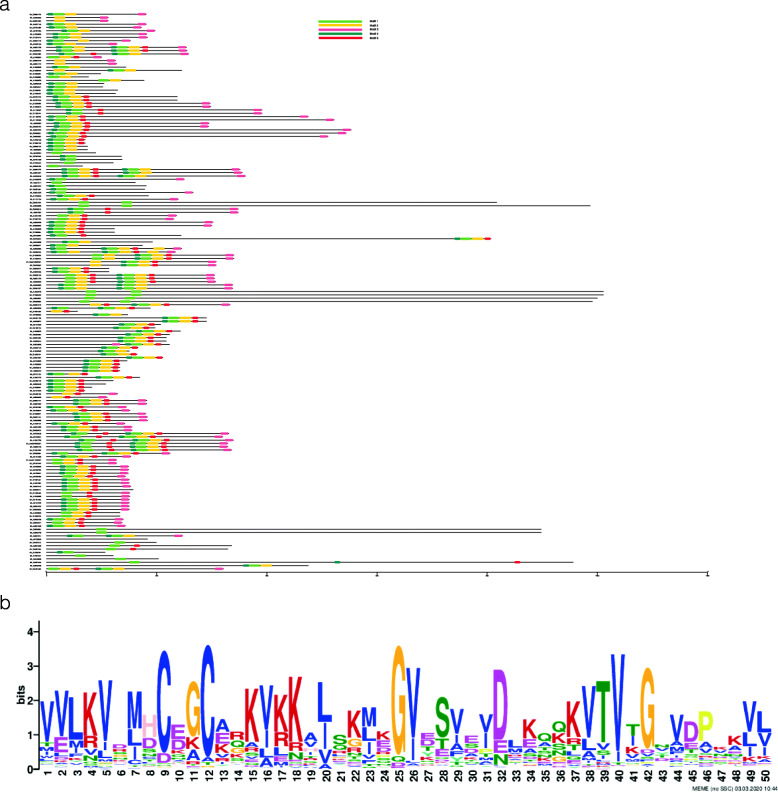
Logo of conserved motifs in HMAD domain in *G. hirsutum*. a: Conserved motifs were predicted from MEME (http://meme-suite.org/tools/meme). The length of proteins were estimated using the scale at the bottom. The motifs were displayed in the different colored boxes with various numbers, black line indicated the non-conserved amino acid. b: Logo of conserved motif was predicted from MEME (http://meme-suite.org/tools/meme)

To investigate the presence of domain sequence and the degree of conservation of the *HMAD* domain in *G. hirsutum*, we performed multiple sequence alignment by using the full-length sequences of the *HMAD* family proteins. The result of different *HMAD* protein groups indicated that five conserved motifs were identified in the sequences of *HMAD* family proteins, and the order of motifs on each family protein was not exactly the same (Fig. 5a). In addition, we also analyzed the conserved *HMAD* domain in all family proteins by multiple sequence alignment, and found a highly conserved motif presence in the domain (Fig. 5b), in which, an anion binding box (CXXC) exist in the catalytic center. Consistent with previous studies [122, 123], the anion binding box with two conserved cysteines as the metal binding.

Based on the Ka/Ks ratio, it could be assumed that Darwinian positive selection was linked with the *HMAD* gene divergence after duplication [124, 125]. In our study, we found that 79 genes pairs had low Ka/Ks ratios (smaller than 0.5) and 24 gene pairs had the Ka/Ks ratios between 0.5 and 1.0. And 13 genes pairs had Ka/Ks larger than 1, might be due to relatively rapid evolution following duplication (Table 2). As most of the Ka/Ks ratios were smaller than 1.0, we presumed that the cotton *HMAD* gene family had undergone strong purifying selection pressure with limited functional divergence that occurred after segmental duplications and whole genome duplications (WGDs).

### Expression profile of *HMAD* genes across different tissues and different stress conditions in TM-1

To understand the temporal and spatial expression patterns of different *HMAD* genes, publicly deposited RNA-seq data was used to assess the expression profile across different tissues (root, stem, leaf, torus, petal, stamen, pistil, calycle), developmental stages (−3dpa ovule, −1dpa ovule, 0dpa ovule, 1dpa ovule, 3dpa ovule, 5dpa ovule, 10dpa ovule, 20dpa ovule, 25dpa ovule, 35dpa ovule, 5dpa fiber, 10dpa fiber, 20dpa fiber, 25dpa fiber) and stresses treatment (1 h treated with cold, 3 h treated with cold, 6 h treated with cold, 12 h treated with cold, 1 h treated with hot, 3 h treated with hot, 6 h treated with hot, 12 h treated with hot, 1 h treated with salt, 3 h treated with salt, 6 h treated with salt, 12 h treated with salt, 1 h treated with PEG, 3 h treated with PEG, 6 h treated with PEG, 12 h treated with PEG). Results showed that the 169 genes can be divided into 10 groups, which include cluster 1 with two genes (*Gh_A08G1780*, *Gh_D08G2126*), cluster 2 with two genes (*Gh_A05G3446*, *Gh_D04G0145*), cluster 3 with just one gene (*Gh_A01G1576*), cluster 4 with two genes (*Gh_D01G1883*, *Gh_D12G1886*), cluster 5 with just one gene (*Gh_D03G0414*), cluster 6 with just one gene (*Gh_D09G0521*), cluster 7 with three genes (*Gh_A01G1399*, *Gh_D01G1640* and *Gh_Sca011408G01*), cluster 8 with two genes (*Gh_D04G0001*, *Gh_Sca013298G01*), cluster 9 with four genes (*Gh_A12G2296*, *Gh_D10G2047*, *Gh_D12G2433* and *Gh_D12G2434*), cluster 10 with 151 genes (Fig. S3).

**Fig. 6:**
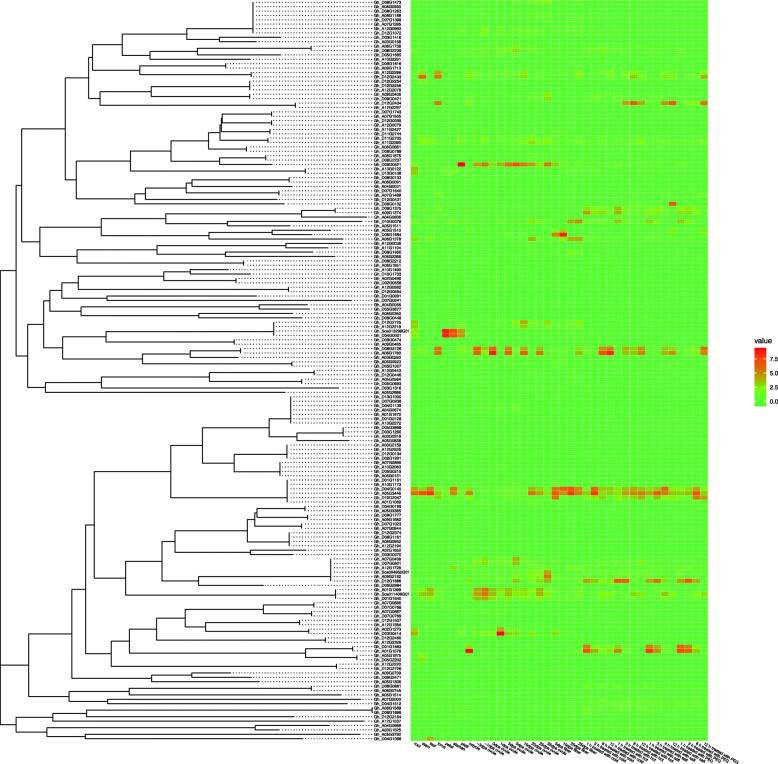
Expression levels of *HMAD* genes in different tissues and different stress. A phylogenetic tree at left from HMAD protein sequences was constructed with MEGA X using neighbor-joining method. The heatmap at right was generated on the basis of RNA-seq data from the website (http://structuralbiology.cau.edu.cn/gossypium/). The color bar represents the expression values. The color scale was shown at the right of the figure. Higher expression levels were shown in red, and lower in green

In cluster 1, the expression level was higher in torus, ovule development every once day, 25dpa fibers and stresses treatment after 6 h. In cluster 2, the expression level was all high (except petals, −3dpa ovule, −1dpa ovule, 0dpa ovule, 1dpa ovule, 3dpa ovule, 5dpa ovule, 10dpa ovule). In cluster 3, the expression level was higher in calycle tissue and stresses treatment after 1 h, which decreased gradually. In cluster 4, the expression level was higher in calycle tissue, 1 h treated with cold, 1 h treated with hot, 1 h treated with salt, 3 h treated with salt, 1 h treated with PEG, 3 h treated with PEG. In cluster 5, the expression level was higher in root tissue and 1dpa ovule. In cluster 6, the expression level was higher in pistil tissue and ovule development especially at 3dpa, 5dpa and 35dpa. In cluster 7, the expression level was higher in leaf tissue and ovule development especially at -1dpa ovule. In cluster 8, the expression level was higher in root, petal, stamen and pistil. In cluster 9, the expression level was higher in torus tissue, calycle, 6 h treated with hot, 6 h treated with salt and 12 h treated with PEG. In cluster 10, there was the largest number of 151 genes, but most of whose expression level were low or even none. While some genes expression level is different, for example, *Gh_D05G1684* highly expressed in the 10dpa in fiber. Interestingly, we found that some *HMAD* genes highly expressed under stress condition (Fig. 6). For example, *Gh_D08G0132* and *Gh_A05G1510* highly expressed after 12 h of the salt stress condition, while *Gh_A01G1576* highly expressed after 1 h of the stress condition (cold, salt, PEG). *Gh_A09G1374*, *Gh_D09G1375*, *Gh_D10G0078* expression level increased under stress condition (cold, salt, PEG).

### Core promoter element analysis

**Fig. 7:**
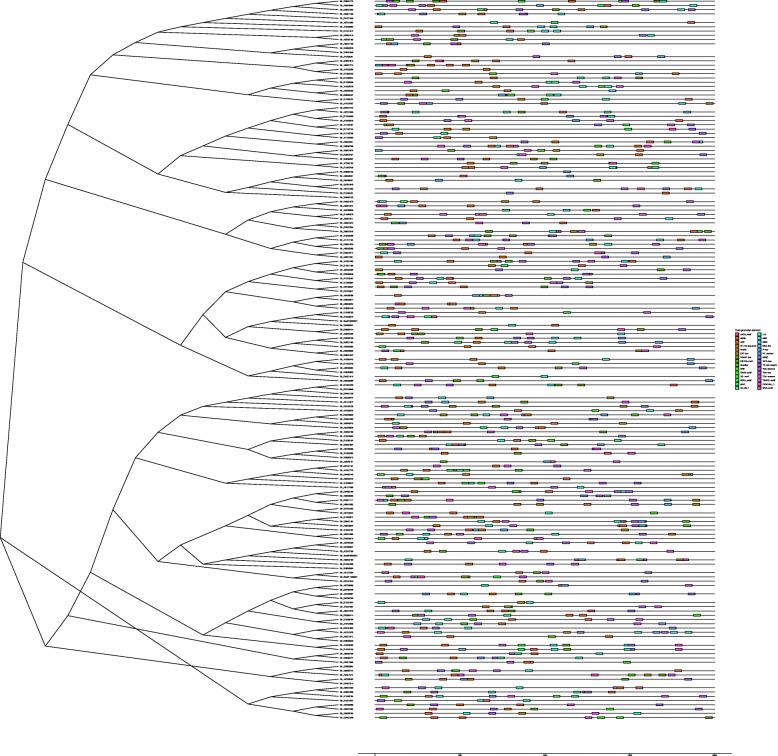
Core promoter element. A phylogenetic tree at left from HMAD protein sequences was constructed with MEGA X using neighbor-joining method. The core promoter element at right were generated based on Plant CARE database (http://bioinformatics.psb.ugent.be/webtools/plantcare/html/). AACA_motif: involved in endosperm-specific negative expression.  ABRE: cis-acting element involved in the abscisic acid responsiveness.  ARE: cis-acting regulatory element essential for the anaerobic induction.  AT−rich sequence: element for maximal elicitor-mediated activation (2copies).  AuxRE: part of an auxin-responsive element.  CAT−box: cis-acting regulatory element related to meristem expression.  CCAAT−box: MYBHv1 binding site.  CGTCA−motif: cis-acting regulatory element involved in the MeJA-responsiveness.  Circadian: cis-acting regulatory element involved in circadian control.  DRE: cis-acting element involved in dehydration, low-temp, salt stresses.  GARE−motif: gibberellin-responsive element.  GC − motif: enhancer-like element involved in anoxic specific inducibility.  GCN4 − motif: cis-regulatory element involved in endosperm expression.  HD − Zip 1: element involved in differentiation of the palisade mesophyll cells.  LTR: cis-acting element involved in low-temperature responsiveness.  MBS: MYB binding site involved in drought-inducibility.  MBSI: MYB binding site involved in flavonoid biosynthetic genes regulation.  MSA − like: cis-acting element involved in cell cycle regulation  P − box: gibberellin-responsive element.  RY − element: cis-acting regulatory element involved in seed-specific regulation.  SARE: cis-acting element involved in salicylic acid responsiveness.  TATC−box: cis-acting element involved in gibberellin-responsiveness.  TC − rich repeats: cis-acting element involved in defense and stress responsiveness.  TCA − element: cis-acting element involved in salicylic acid responsiveness.  TGA − box: part of an auxin-responsive element.  TGA − element: auxin-responsive element.  TGACG−motif: cis-acting regulatory element involved in the MeJA-responsiveness.  Unnamed_1: 60 K protein binding site.  WUN − motif: wound-responsive element

To further explore why *HMAD* gene family highly expressed under biotic stress condition except heavy metal, the core promoter element of *HMAD* genes from *G. hirsutum* were divided into four types (hormone, stress, tissue and others) (Fig. 7), among which, element involved in hormone-responsiveness mainly contained ABA (abscisic acid), GA (gibberellins), IAA/auxin, SA (salicylic acid), MeJA (Methyl jasmonate). Element involved in defense and stress responsiveness mainly contained drought, low-temperature, dehydration, salt stress, anaerobic, among which, 72 genes involved in drought, 51 genes involved in low-temperature responsiveness, 55 genes involved in defense and stress responsiveness with TC-rich repeats element, and 1 gene (*Gh_D04G1066*) both involved in salt and low-temperature responsiveness. In total, there were 111 genes of 169 *HMAD* genes with core promoter element responding to stress. As described above in TM-1 RNA-seq data, 12 of the 18 genes were highly expressed with at least one abiotic stress-related promoter element (Table S2). Element involved in tissues including the palisade mesophyll cells, meristem, endosperm, seed-specific. And element involved in other’s function, such as circadian control, cell cycle, flavonoid biosynthetic. It was interesting that 9 of 12 genes with element of flavonoid biosynthetic were along with other’s stress element. In previous study, anthocyanins, as secondary metabolites, may respond to stress resistance through osmotic equilibrium [126–128]. For example, *Gh_A01G1576* highly expressed after 1 h of the stress condition (cold, salt, PEG), whose core promoter element contained drought-inducibility, low-temperature responsiveness and MBSI promoter element involved in flavonoid biosynthetic genes regulation (Table S2).

### The expression level of *HMAD* gene in different tissues under Na_2_SO_4_ stress

**Fig. 8:**
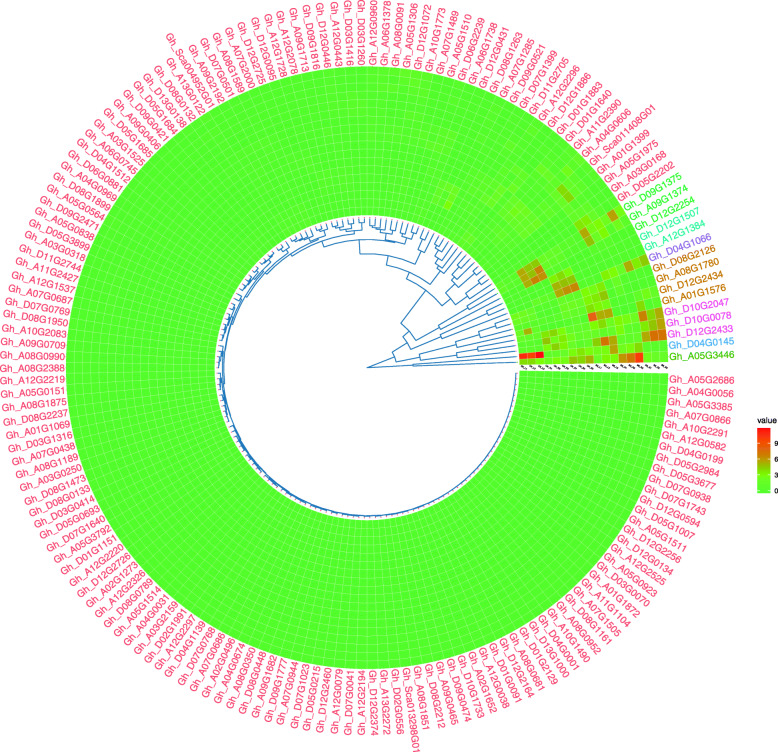
The transcriptome expression levels of *HMAD* genes in different tissues (root, stem, leaf) between control and treatment with 300 mM Na_2_SO_4_. The color bar represents the expression values. The color scale was shown at the right of the figure. Higher expression levels were shown in red, and lower in green

To identify the function of *HMAD* genes under other abiotic stress, we used the material Zhong 9835 [129]. Based on the *HMAD* gene family of RNA-seq data (Fig. 8) in Zhong 9835 (Table S5), 14 genes significantly expressed differentially in roots, stems and leaves between control and treatment with 300 mM Na_2_SO_4_ (Table S4, Fig. S1), in which 10 genes with at least one core promoter element about stress (Table S2). It is interesting to note that 3 of 4 flavonoid biosynthetic element were along with the stress element. More important, some genes highly expressed in both TM-1 and Zhong 9835 under stress condition, such as *Gh_D04G0145*, *Gh_D10G0078*, *Gh_Sca011408G01*, *Gh_A01G1576* and so on.

## Discussion

In this study, *HMAD* family genes from *G.arboreum* (84 genes), *G. raimondii* (76 genes), *G. hirsutum* (169 genes), and *G. barbadense* (159 genes), respectively were identified, which contain the total numbers of *HMAD* genes in the two diploid cotton (*G. arboreum* and *G. raimondii*), as A and D genome donor species, were lower than that in allotetraploid (*G. hirsutum* and *G. barbadense*) cotton. Syntenic analysis of the *HMAD* gene family in four cotton species revealed that 4 genes in *G. arboreum* and 11 genes in *G. raimondii* were lost during evolution, while 24 genes appeared in *G. hirsutum*, showing that these genes played a critical role in cotton evolution. As most of the Ka/Ks ratios were smaller than 1.0, we presumed that the cotton *HMAD* gene family underwent strong purifying selection pressure with limited functional divergence. These results suggested that there was possible gene loss and/or as a result of chromosome rearrangement during the evolution [121].

169 *G. hirsutum* genes were identified belonging to the *HMAD* gene family. The molecular weights (kDa) of 169 *HMAD* proteins ranged from 5.8 to 108.5 kDa (Table 1). The isoelectric point (pI) of the majority of the 169 *HMAD* proteins was alkaline except for 55 genes less than 7.6 (Table 1). The various molecular weight and gene sequence length indicated that the physical and chemical properties of *HMAD* family genes have little difference. Based on the WoLF PSORT analysis, the *HMAD* family genes are mainly distributed in the chloroplast (62 genes), the cytosol (54 genes), the nucleus (39 genes) and the plasma membrane (11 genes) (Table 1). 169 *HMAD* genes were divided into five subclasses: I, II, III, IV, Va, Vb, among which the II subclass contained the highest number of genes (66 members) and followed by III subclass (29 members). Structural analysis of the 169 *HMAD* gene family showed that just 5 genes (*Gh_D01G1640*, *Gh_Sca011408G01*, *Gh_A05G3385* of group I, *Gh_D08G1263* and *Gh_A08G0990* of group Vb) contained no intron. While the rest of the *HMAD* genes contain multiple introns, especially P1B-ATPases *HMA5–8* contains most introns than other genes. Among the gene functional annotations of 169 *HMAD* genes, the number of Heavy metal transport/detoxification superfamily proteins is 116 (Table S5), which are divided into four categories between the phylogeny tree and gene structural. 13 genes pairs had Ka/Ks larger than 1, which includes 13 Heavy metal transport/detoxification superfamily proteins (*Gh_A05G2686*, *Gh_A08G1875*, *Gh_A10G1490*, *Gh_A10G2083*, *Gh_A11G1104*, *Gh_A12G0038*, *Gh_A12G0079*, *Gh_A13G2272*, *Gh_D05G0215*, *Gh_D05G2984*, *Gh_D07G0938*, *Gh_D08G2237*, *Gh_D10G1733*). Additionally, the signature of four conserved amino acids CXXC for binding metal ions was discovered through sequence alignment [54, 55]. The classified genes and conserved motifs with conserved amino acids CXXC for binding metal ions indicated that 169 *HMAD* genes may be different response to heavy metals in various organelles, especially some Heavy metal transport/detoxification superfamily proteins under relatively rapid evolution.

Gene expression patterns with the differentiation of promoter regions can provide important insights to gene function [130]. After the RNA-seq data of TM-1 analysis, the most genes of expression level cluster 10 with 151 genes had a lower expression level or none. And after the promoter element analysis of four types (hormone, stress, tissue and others), there were 111 genes of 169 *HMAD* genes with core promoter element responding to stress. The results showed that 169 *HMAD* genes were not widely expressed in tissues as well as under stress condition (cold, salt, PEG) (Fig. 6), indicating their critical role in different tissues and stress condition with different promoter elements.

Cotton is half halophytes, and Zhong 9835 was resistance to salt [131], including Na_2_SO_4_. Based on the transcriptome data of TM-1, we found that heavy metal transport protein highly expressed under adversity abiotic stress condition. Further, through gene sequences and promoter element analysis, we found that *HMAD* evolution speed was quickly, which divided into five types of *HMAD* family, and some of those genes with responding to stress element had a highly expression under adversity abiotic stress condition. According to the analysis of the root, stem and leaf between Na_2_SO_4_ treatment and control, 14 genes with stress element significantly expressed differentially (Fig. S1). *HMAD* highly expressed under salt condition, probably because of ROS caused by ion balance [6]. For example, on the one hand, gene expression in ROS way and ion balance maintenance, such as Ca^2+^ signaling pathway and MAPK, MYB transcription factor [132, 133], programmed cell death [134, 135]. And then the GSH, as the main way to remove ROS under the condition of high concentration, can not only response to heavy metal ions [136], also can response to salt stress ion [137]. At last, the balance of ions, such as anthocyanins were associated with the salt stress [6]. *HMAD* with anthocyanins related promoter elements highly expressed under Na_2_SO_4_ condition, similar to previous study that anthocyanins involved in resistance to salt, at the same time involved in heavy metal transport [137]. On the other hand, the transfer of heavy metals and salt stress are vacuole segregation [138, 139], such as the P-type ATP as an important role, can not only balance the salt ions and also can balance of heavy metal ions [140, 141].

In additional, *HMA* genes can selectively absorb and transport metal ions [142]. CtpB, as a plasma membrane copper (I) transporting P-type ATPase of *Mycobacterium tuberculosis*, is different from copper detoxification [143]. In *Mycobacterium tuberculosis*, Cu^2+^, Co^2+^, Ni^2+^, Zn^2+^, Cd^2+^ and Pb^2+^ stimulate the ATPase activity of the putative P1B-type ATPase CtpG in the plasma membrane, while Cd^2+^ more efficiently than other heavy metal cations across the mycobacterial plasma membrane [144]. Chaperone is an important way in delivering Cu to heavy metal P1B-ATPases [143]. In general, *HMA* contain approximately 6–8 transmembrane helices, a soluble nucleotide binding domain, phosphorylation domain, and a soluble actuator domain, of which *HMA1–4* belonging to Zn^2+^/Co^2+^/Cd^2+^/Pb^2+^ transporting, although *HMA1* conserved amino acids is different from the *HMA2*, *HMA3* and *HMA4* [143], whereas *HMA5–8* belong to the Cu^+^/Ag^+^ subclass [144].

The sequences of *HMA* (Heavy Metal ATPase) of P1B-ATP from *G. hirsutum* based on the sequences of *HMA* in *A. thaliana*, also contained P-ATPases (E1-E2 ATPases) and *HAD* (halo acid dehydrogenase) domain and *HMAD* (heavy-metal-associated domain) domain (Table S3). In this study, *HMAD* gene family contained *HMA5*-*HMA8* (except *Gh_A08G2387*) (Table S3). *HMA5* localized in the plasma membrane, of which *Gh_A05G0564*, *Gh_A08G2388*, *Gh_D05G0693* with 8 TMHs, while *Gh_D08G1950* with 6 TMHs. In *HMA6*, *Gh_A03G1525* with 7 TMHs localized in the plasma membrane, whereas *Gh_A04G0969* and *Gh_D04G1512* without TMHs localized in the chloroplast. *HMA7* and *HMA8* localized in the plasma membrane with 8 TMHs and 5 TMHs, respectively. Obviously, in cotton *HMA* genes evolutionarily adapted quickly in the TM region through the analysis of the sequence, gene structure, Ka/Ks ratio and the phylogenetic tree [144].

## Conclusions

In summary, we identified 169, 159, 76 and 84 full-length putative *HMAD* genes in *G. hirsutum*, *G. barbadense*, *G. arboreum* and *G. raimondii,* which were much larger than that of the other gene families. We also found that *HMAD* gene family with promoter elements in response to stress, may plays important roles in different abiotic stress. Our results provided a foundation to further explore the crosstalk of molecular mechanism of *HMAD* genes under abiotic stress and heavy metal condition.

## Methods

### Cotton genome and RNA-seq resources

The sequenced genome data and annotation information of four *Gossypium* species including *G. raimondii*, (JGI_v2) *G. arboreum*, (CRI_v1.1) *G. hirsutum* (NAU-NBI_v1.1) and *G. barbadense* (ZJU_v1.1) were downloaded from the Cottongen (https://www.cottongen.org/). RNA-seq data for gene expression analysis in *G. hirsutum* was downloaded from ccNET database (http://structuralbiology.cau.edu.cn/gossypium/), which mainly includes the gene expression data under some stress conditions available in the BioProject database under accession no. PRJNA248163, such as root (SRR1695173), stem (SRR1695174), leaf (SRR1695175), torus (SRR1695176), petal (SRR1695177), stamen (SRR1695178), pistil (SRR1695179), calycle (SRR1695180), −3dpa ovule (SRR1695181), −1dpa ovule (SRR1695182), 0dpa ovule (SRR1695183), 1dpa ovule (SRR1695184), 3dpa ovule (SRR1695185), 5dpa ovule (SRR1695186), 10dpa ovule (SRR1695187), 20dpa ovule (SRR1695188), 25dpa ovule (SRR1695189), 35dpa ovule (SRR1695190), 5dpa fiber (SRR1695191), 10dpa fiber (SRR1695192), 20dpa fiber (SRR1695193), 25dpa fiber (SRR1695194), 1 h treated with cold (SRR1768504), 3 h treated with cold (SRR1768505), 6 h treated with cold (SRR1768506), 12 h treated with cold (SRR1768507), 1 h treated with hot (SRR1768508), 3 h treated with hot (SRR1768509), 6 h treated with hot (SRR1768510), 12 h treated with hot (SRR1768511), 1 h treated with salt (SRR1768512), 3 h treated with salt (SRR1768513), 6 h treated with salt (SRR1768514), 12 h treated with salt (SRR1768515), 1 h treated with PEG (SRR1768516), 3 h treated with PEG (SRR1768517), 6 h treated with PEG (SRR1768518), 12 h treated with PEG (SRR1768519). The raw RNA-Seq data of Zhong 9835, a preserved self-bred line from cultivar of *G. hirsutum*, about Na_2_SO_4_ tolerance generated in this study are available in the BioProject database under accession no. PRJNA703009.

### Identification of HMAD domain-containing genes

To identify the HMAD domain-containing genes, the hidden Markov Models (HMM) of HMAD domain (PF00403) was downloaded from Pfam 29.0 database (http://pfam.xfam.org/), then HMMER 3.0 software was used to retrieve the whole genome database of four cotton species by [145] and further identified gene family by pfamscan website (https://www.ebi.ac.uk/Tools/pfa/pfamscan/) and (http://smart.emblheidelberg.de/) SMART (Simple Modular Architecture Research Tool) for confirmation of results. The redundant sequences without HMAD domain were manually checked and then removed. Molecular weight (MW), theoretical isoelectric point (pI), Signal peptide and size of the HMAD were investigated with the online tool ExPASy (http://expasy.org/tools/). Subcellular locations were predicted by software WoLF PSORT (http://wolfpsort.org/). The putative transmembrane helixes were also predicted using TMHMM Server V.2.0 (http://www.cbs.dtu.dk/services/TMHMM/).

### Phylogenetic analysis

The multiple sequence alignment of HMAD domain sequence containing genes of four cotton species was accomplished by ClustalX2 software [146] with default parameters. The unrooted phylogenetic tree was constructed by the neighbour joining tree (NJ) in MEGA X software [147] (https://www.megasoftware.net/) with the bootstrap analysis for 1000 iterations and ggtree (v2.2.4) packages [148] of R (v4.0.3) software.

### Chromosomal mapping and gene duplication

The physical location data of *HMAD* genes were retrieved from genome sequence data of four cotton species, and was subsequently used to map these genes using Mapchart-2.23 [149]. Synonymous and non-synonymous rates of evolution were computed using the maximum likelihood method by the Ka/Ks calculator 2.0 [150].

### Gene structure and domain analysis

The exon and intron organizations of *HMAD* genes inferred in the gene structure display server (http://gsds.cbi.pku.edu.cn/) through comparison of genomic and CDS sequences. The conserved motifs in *HMAD* genes were identified by MEME (http://meme-suite.org/tools/meme) and TBtools-0.6673 [151].

### Genome wide synteny analysis of *HMAD* genes

A BLASTP comparison was used to obtain the pair wise gene information between two allotetraploid cotton species (*G. hirsutum* and *G. barbadense*) and two diploid cotton species (*G. raimondii* and *G. arboreum*). According to the BLASTP output, the synteny analysis was constructed using circos-0.69-3 software package (http://circos.ca/software/) with default parameters.

### Analysis of cis-elements in the promoters

Promoter element sequences extracted from upstream 2000 bp of genes, cis-element were found through Plant CARE database (http://bioinformatics.psb.ugent.be/webtools/plantcare/html/).

### RNA-seq between control and treatment with Na_2_SO_4_

Zhong 9835, a preserved self-bred line from cultivar of *G. hirsutum* by our lab, was used for this study. Seeds were sown in sand soil pots. The sand was washed cleanly and sterilized at 121 °C for 8 h. Four seedlings in each pot were cultivated in a 28 °C/16 h light and 25 °C/8 h dark cycle with a light intensity of 150 μmol·m-2·s-1 and 75% relative humidity for approximately 30 days. Then, 300 mM Na_2_SO_4_ after 12 h was chosen as the applicable stress concentration and time. Seedlings transplanted into normal water were used as controls. After exposure for 12 h, leaf, stem and whole root samples were collected. Each sample was tested three times. Samples were frozen in liquid nitrogen and stored at − 80 °C.

### RNA extraction and qRT-PCR analysis

Total RNA was isolated from root, stem and leaf between control and treatment with 300 mM Na_2_SO_4_ in the Zhong 9835 by the EASY spin Plant RNA Kit (TIANGEN). Afterwards, first-strand cDNA was synthesized using Prime Script TM II 1st strand cDNA Synthesis Kit (TaKaRa) according to the manufacturer’s instructions. The qRT-PCR was carried out in 20 μL volume containing 1.4 μL cDNA, 0.8 μL of 10 μM forward and reverse primer, 10 μL SYBR Premix Ex Taq II (2×), and 7.8 μL ddH_2_O. PCR amplification was performed under the denaturation at 95 °C for 30 s; 40 cycles at 95 °C for 5 s and 60 °C for 30 s; followed by 95 °C for 15 s, 60 °C for 1 min by Bio-Rad CFX96 Real-Time PCR system. qRT-PCR was carried out by the gene-specific primers, Histone3 (AF024716) (F: TCAAGACTGATTTGCGTTTCCA, R: GCGCAAAGGTTGGTGTCTTC) was employed as an internal control. In the end, relative gene expression was quantified using the 2^–△△Ct^ method.

## Supplementary Information

**Additional file 1 **: **Fig. S1.** Expression profiles of qRT-PCR of *HMAD* genes between control and treatment with 300 mM Na_2_SO_4_ among root, stem and leaf. The expression patterns of 31 *HMAD* genes in Zhong 9835 between control and treatment. qRT-PCR was conducted to analyze the relative expression of 31 *HMAD* genes in root, stem, leaf. R represents root, S represents stem, L represents leaf. CK_R represents root with control, SS_R represents root with treatment. CK_S represents stem with control, SS_S represents stem with treatment. CK_L represents leaf with control, SS_L represents leaf with treatment.

**Additional file 2 **: **Fig. S2.** The synteny and collinearity analysis of *HMAD* genes between *G.barbadense* and *G.hirsutum*. A01-A13 represented the chromosomes from At sub genome while D01-D13 represented the chromosomes from Dt sub genome. The Arabic numerals (1–3) at *G.hirsutum* in bars represented scaffold4952, scaffold11408, scaffold13298, respectively.

**Additional file 3 **: **Fig. S3.** Expression levels of *HMAD* genes in different tissues and different stress. The heatmap was generated on the basis of RNA-seq data from the website (http://structuralbiology.cau.edu.cn/gossypium/). The color bar represents the expression values. The color scale was shown at the right of the figure. Higher expression levels were shown in red, and lower in green.

**Additional file 4 **: **Table S1.** The information of HMAD proteins in four *Gossypium* spp.

**Additional file 5 **: **Table S2.** Analysis of high-expression gene with cis-elements.

**Additional file 6 **: **Table S3.** The information of P1B-ATPases proteins between *Gossypium_hirsutum* and *A. thaliana*.

**Additional file 7 **: **Table S4.** Primers of qRT-PCR used in this study.

**Additional file 8 **: **Table S5.** The information of transcriptome data of *HMAD* genes between control and treatment with 300 mM Na_2_SO_4_.

## Data Availability

All of the data and materials supporting our research findings are contained in the methods section of the manuscript. Details are provided in the attached Additional files. The datasets generated and/or analysed during the current study are available in the NCBI repository [PRJNA248163 and PRJNA703009].

## Abbreviations

HMAD: Heavy-metal-associated domain
HMA: Heavy metal ATPase
DPA: Days post anthesis
MAPK: Mitogen activated protein kinase
PCs: Phytochelatins
MTs: Metallothioneins
ABC: ATP-binding cassette
NRAMP: Natural resistance and macrophage protein
CDF: Cation Diffusion Facilitator
YSL: Yellow-stripe-like
HMA4: Heavy metal ATPase 4
IRT2: Iron-regulated protein 2
WGDs: Whole genome duplications
