# Supplementary figures and images for: Genome-wide identification and function analysis of HMAD gene family in cotton (Gossypium spp.)
Source: BMC Plant Biol. 2021 Aug 20;21:386. doi: 10.1186/s12870-021-03170-8 (PMC8377987; doi:10.1186/s12870-021-03170-8)

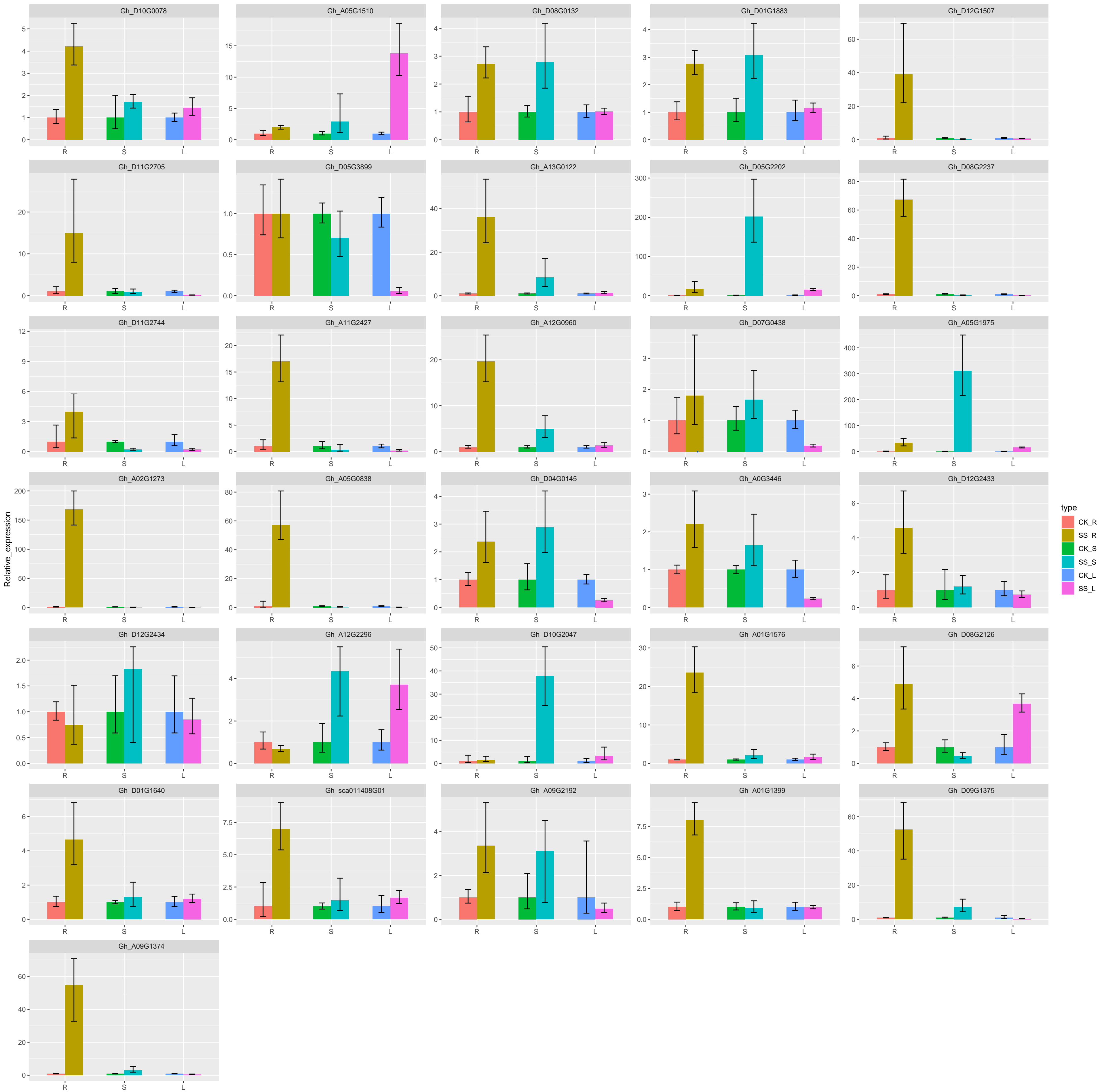

Supplement: Supplementary file 1 — Additional file 1 : Fig. S1. Expression profiles of qRT-PCR of HMAD genes between control and treatment with 300 mM Na2SO4 among root, stem and leaf. The expression patterns of 31 HMAD genes in Zhong 9835 between control and treatment. qRT-PCR was conducted to analyze the relative expression of 31 HMAD genes in root, stem, leaf. R represents root, S represents stem, L represents leaf. CK_R represents root with control, SS_R represents root with treatment. CK_S represents stem with control, SS_S represents stem with treatment. CK_L represents leaf with control, SS_L represents leaf with treatment. [file 12870_2021_3170_MOESM1_ESM.pdf]

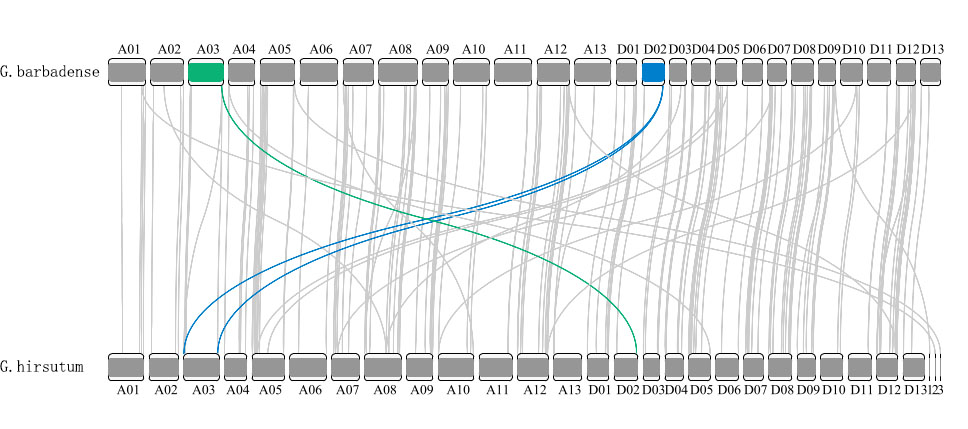

Supplement: Supplementary file 2 — Additional file 2 : Fig. S2. The synteny and collinearity analysis of HMAD genes between G.barbadense and G.hirsutum. A01-A13 represented the chromosomes from At sub genome while D01-D13 represented the chromosomes from Dt sub genome. The Arabic numerals (1–3) at G.hirsutum in bars represented scaffold4952, scaffold11408, scaffold13298, respectively. [file 12870_2021_3170_MOESM2_ESM.jpg]

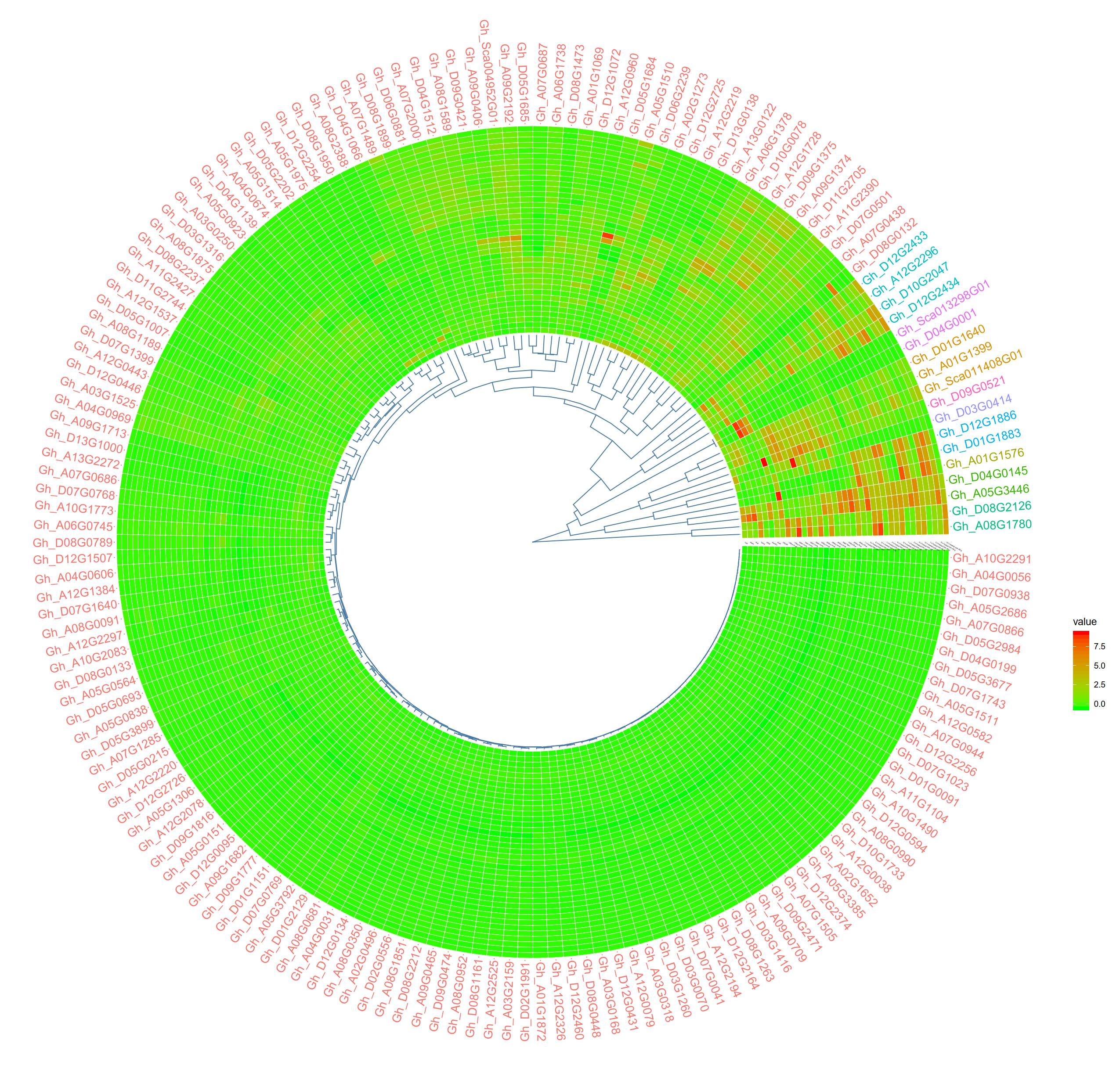

Supplement: Supplementary file 3 — Additional file 3 : Fig. S3. Expression levels of HMAD genes in different tissues and different stress. The heatmap was generated on the basis of RNA-seq data from the website (http://structuralbiology.cau.edu.cn/gossypium/). The color bar represents the expression values. The color scale was shown at the right of the figure. Higher expression levels were shown in red, and lower in green. [file 12870_2021_3170_MOESM3_ESM.tif]
